# GIT1 promotes lung cancer cell metastasis through modulating Rac1/Cdc42 activity and is associated with poor prognosis

**DOI:** 10.18632/oncotarget.5531

**Published:** 2015-10-08

**Authors:** Jeng-Shou Chang, Chia-Yi Su, Wen-Hsuan Yu, Wei-Jiunn Lee, Yu-Peng Liu, Tsung-Ching Lai, Yi-Hua Jan, Yi-Fang Yang, Chia-Ning Shen, Jin-Yuh Shew, Jean Lu, Chih-Jen Yang, Ming-Shyan Huang, Pei-Jung Lu, Yuan-Feng Lin, Min-Liang Kuo, Kuo-Tai Hua, Michael Hsiao

**Affiliations:** ^1^ Institute of Biochemistry and Molecular Biology, National Yang-Ming University, Taipei, Taiwan; ^2^ Medical Biology, Genomics Research Center, Academia Sinica, Taipei, Taiwan; ^3^ Department of Molecular and Cellular Oncology, The University of Texas MD Anderson Cancer Center, Houston, Texas, USA; ^4^ Graduate School of Biomedical Sciences, The University of Texas Houston Health Science Center, Houston, Texas, USA; ^5^ Department of Urology, School of Medicine, College of Medicine, Taipei Medical University, Taipei, Taiwan; ^6^ Department of Medical Education and Research, Wan Fang Hospital, Taipei Medical University, Taipei, Taiwan; ^7^ Institute of Genomic Medicine, Kaohsiung Medical University, Kaohsiung, Taiwan; ^8^ Department of Internal Medicine, Kaohsiung Medical University Hospital, Kaohsiung Medical University, Kaohsiung, Taiwan; ^9^ Institute of Clinical Medicine, Medical College, National Cheng Kung University, Tainan, Taiwan; ^10^ Graduate Institute of Clinical Medicine, College of Medicine, Taipei Medical University, Taipei, Taiwan; ^11^ Institute of Biochemical Science, National Taiwan University College of Life Science, Taipei, Taiwan; ^12^ Graduate Institute of Toxicology, National Taiwan University College of Medicine, Taipei, Taiwan

**Keywords:** GIT1, Rac1/Cdc42, Rho GTPases, metastasis, lung cancer prognosis

## Abstract

G-protein-coupled receptor kinase interacting protein 1 (GIT1) is participated in cell movement activation, which is a fundamental process during tissue development and cancer progression. GIT1/PIX forming a functional protein complex that contributes to Rac1/Cdc42 activation, resulting in increasing cell mobility. Although the importance of Rac1/Cdc42 activation is well documented in cancer aggressiveness, the clinical importance of GIT1 remains largely unknown. Here, we investigated the clinical significance of GIT1 expression in non-small-cell lung cancer (NSCLC) and also verified the importance of GIT1-Rac1/Cdc42 axis in stimulating NSCLC cell mobility. The result indicated higher GIT1 expression patients had significantly poorer prognoses in disease-free survival (DFS) and overall survival (OS) compared with lower GIT1 expression patients. Higher GIT1 expression was an independent prognostic factor by multivariate analysis and associated with migration/invasion of NSCLC cells in transwell assay. *In vivo* studies indicated that GIT1 promotes metastasis of NSCLC cells. Finally, GIT1 was found to stimulate migration/invasion by altering the activity of Rac1/Cdc42 in NSCLC cells. Together, the GIT1 expression is associated with poor prognosis in patients with NSCLC. GIT1 is critical for the invasiveness of NSCLC cells through stimulating the activity of Rac1/Cdc42.

## INTRODUCTION

Lung cancer is the leading cause of cancer-related deaths worldwide [1]. Non-small cell lung cancer (NSCLC) accounts for 80% of all lung cancer cases, and the 5-year survival rate of patients with stage IV NSCLC is <1% [2, 3]. Despite advances in treatment modalities, including surgical resection, radiation therapy, chemotherapy, target therapy, and combinations of these therapies, the prognosis of early NSCLC remains poor. Therefore, characterization and identification of novel prognostic markers and therapeutic targets are urgently needed.

G-protein-coupled receptor (GPCR)-kinase interacting protein-1 (GIT1) is a multi-functional protein. GIT1 has multiple domains, including ARFGAP, Spa2 homology, three ankyrin repeat, coiled-coil and paxillin-binding domains, and is known to interact with diverse molecules [4, 5]. It has been reported that GIT1 participates in a wide variety of functions, including GPCR endocytosis, turnover of focal adhesions, spine morphogenesis, synapse formation, cell mobility, VEGF-mediated angiogenesis, centrosome dynamics, and huntingtin aggregation [4, 6–11]. One of the major roles of GIT1 is in the assembly of p21-activated kinase-interacting exchange factors (PIX) with Paxillin in focal adhesions, a key step in the regulation of cell migration. PIX-GIT1-Paxillin is a pro-migratory protein complex and mediates the activation of Rac1 and Cdc42 (members of the Rho family of GTPases) in the focal complexes of leading edges [8]. Previous studies have identified GIT1-regulated cell migration in neurons, fibroblasts, immune cells, and endothelial cells [8, 12–14]. However, the role of GIT1 in cancer invasiveness and metastasis is relatively less understood.

The up-regulation of GIT1 has recently been reported in oral, cervical, breast, liver and colon cancer [15–18]. It has been shown that GIT1 levels were positively correlated with lymph node metastasis in oral squamous cell carcinoma [15]. Moreover, in our previous study, we found that GIT1 is involved in the regulation of lung cancer cell motility [19]. Although correlation between GIT1 and lung cancer cell migration has been observed, the role of GIT1 in NSCLC progression is still elusive. Furthermore, the clinical significance of GIT1 in NSCLC remains unknown.

In this study, we have elucidated the important influence GIT1 has on the prognosis and clinicopathological characteristics of NSCLC patients. We have also verified the importance of the GIT1-Rac1/Cdc42 axis in facilitating invasion and metastasis of NSCLC cells.

## RESULTS

### GIT1 overexpression correlates with poor prognosis in NSCLC tumors

**Figure 1:**
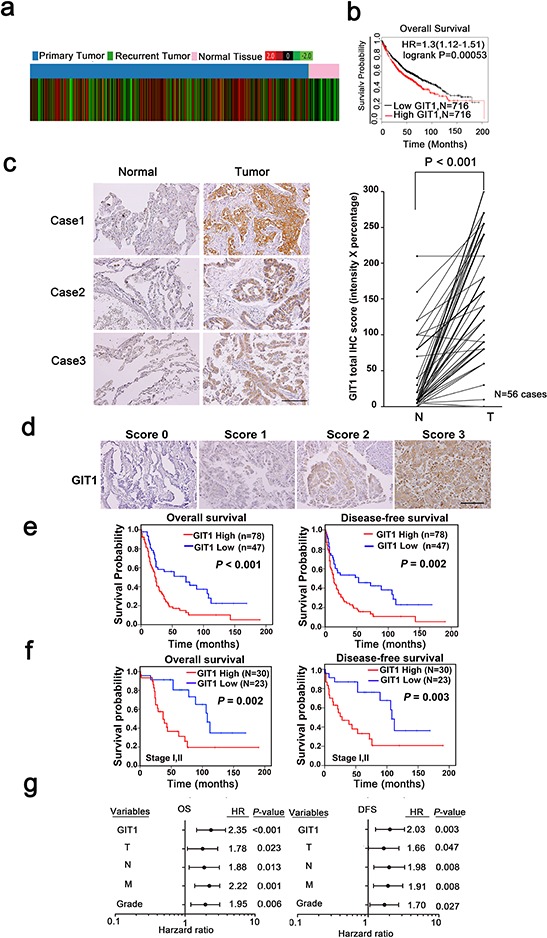
GIT1 overexpression correlates with poor prognosis in NSCLC tumors **a.** Public database analysis of the clinical significance of GIT1 mRNA expression in lung cancer using TCGA contains 1124 cases. Red color in the heat map indicates high GIT1 expression and green color indicates low GIT1 expression. **b.** In Kaplan-Meier plotter microarray database, overall survival plot shows that patients with high GIT1 expression had poor prognosis. Individual database number and *P* value of each plot are indicated. **c.** Representative images from IHC staining of GIT1 protein levels in matched primary lung tumors and normal adjacent tissues. Scale bars, 100 μm. Quantification of cytoplasmic IHC expression of GIT1 in primary lung tumors in comparison with paired normal tissues. The scores are calculated as staining intensity multiplied by percentage of stained cells. **d.** Scores indicating GIT1 levels in representative lung tumor tissues. Scale bars, 100 μm. **e, f.** Kaplan–Meier plots of overall survival and disease-free survival of 125 patients and early stage (stage I and II) of 53 patients with non-small cell lung cancer stratified by GIT1 level. The differences between groups were tested using log rank tests. **g.** Multivariate regression analysis of TNM prognostic factors and GIT1 expression.

To elucidate the clinical relevance of GIT1 in lung cancer patients, we first analyzed GIT1 mRNA expression profiles from TCGA database. The expression levels of GIT1 mRNA were significantly higher in primary tumors compared with normal lung tissues (Figure 1a, *P* < 0.001). Besides, We also examined the correlation between GIT1 mRNA levels and overall survival among lung cancer patients by using Kaplan-Meier (KM) Plotter [21], an online meta-analysis-based biomarker assessment tool. Among the 1432 lung cancer patients, lower GIT1 mRNA levels were significantly correlated with longer survival periods (Figure 1b). Similar correlations between GIT1 expression and survival of lung cancer patients were also observed in SurvExpress databases [22] (Supplementary Figure S1).

We further analyzed GIT1 protein levels in a cohort of 125 NSCLC specimens using immunohistochemistry (IHC) staining as the training cohort. We compared 56 sets of matched samples from primary lung tumors and normal adjacent tissues in this tissue array. Strikingly, in 53 of 56 patients (~95%), GIT1 protein levels were significantly higher in tumors compared with normal tissues (Figure 1c, *P* < 0.001). Next, we determined whether GIT1 expression in NSCLC was associated with NSCLC patient survival. Representative GIT1 staining patterns in NSCLC tissues of the defined scoring criteria are shown in Figure 1d. Our data show that higher expression of GIT1 (a score of 2 or 3) was significantly correlated with reduced overall survival (Figure 1e, *P* < 0.001) and disease-free survival (*P* = 0.002) compared with patients with lower GIT1 expression (a score of 0 or 1). In addition, we also verified our results in another independent NSCLC cohort, the Korean cohort, which served as the validation cohort (Supplementary Figure S2). Analysis of this cohort also showed that higher expression of GIT1 was significantly correlated with poor prognosis, thus providing further evidence that GIT1 is associated with poor survival in NSCLC patients.

Furthermore, we separated the 125 NSCLC cases into early stage (stages I and II) and late stage (stages III and IV) lung cancer patients. The data indicated that GIT1 expression was significantly correlated with reduced overall survival (*P* = 0.002) and disease-free survival (*P* = 0.003) in early stage patients (Figure 1f and Supplementary Figure S2). We also classified our training cohort to adenocarcinoma (AD), squamous cell carcinoma (SCC) and large cell carcinoma (LCC). The data indicated that GIT1 expression was significantly correlate with overall survival (*P* = 0.002) and disease free survival (*P* = 0.007) of AD, but not SCC and LCC (Supplementary Figure S3a). We next determine whether GIT2, a subfamily member of GIT, sharing 85% similarity with GIT1, also serve as a poor prognosis marker in our training cohort. The result indicated that GIT2 is not significantly correlate with poor survival in NSCLC patient (Supplementary Figure S3b). Taken together, GIT1 expression correlated with poor survival of NSCLC especially in AD.

The clinicopathologic features of 125 NSCLC patients with primary tumors are shown in Supplementary Table S1. Furthermore, in the multivariate survival analysis, GIT1 expression was found to be a strong, independent prognostic predictor of reduced overall survival (OS) (hazard ratio [HR] = 2.35; 95% confidence interval [CI] = 1.46–3.79; *P* < 0.001) and reduced disease-free survival (DFS) (hazard ratio [HR] = 2.03; 95% confidence interval [CI] = 1.27–3.26; *P* = 0.003) in NSCLC patients (Figure 1g and Supplementary Table S2). Similar results were also obtained in the Korean lung cancer cohort used as a validation set (Supplementary Table S3).

**Table 1:** The relationship between GIT1 expression and the clinicopathological characteristics of Non-Small Cell Lung Cancer (NSCLC) in training cohort

Clinicopathological Characteristics	*n*	GIT1 expression, *n* (%)		*P*^a^
		Low (*n* = 47)	High (*n* = 78)	
Age				
<65 y	73	26 (35.6%)	47 (64.4%)	0.587
≥65 y	52	21 (40.7%)	31 (59.3%)	
Gender				
Male	71	25 (35.2%)	46 (64.8%)	0.527
Female	54	22 (40.7%)	32 (59.3%)	
Smoking				
Smoker	50	17 (34.0%)	33 (66.0%)	0.497
Non-smoker	75	30 (40.0%)	45 (60.0%)	
Histology				
Adenocarcinoma	81	34 (42.0%)	47 (58.0%)	0.367
Squamous cell carcinoma	35	10 (28.6%)	25 (71.4%)	
Adenosquamous carcinoma	2	0 (0%)	2 (100%)	
Large cell carcinoma	7	3 (42.9%)	4 (57.1%)	
T stage^b^				
T1+T2	89	32 (36.0%)	57 (64.0%)	0.551
T3+T4	36	15 (41.7%)	21 (58.3%)	
N stage^b^				
N0	48	24 (50.0%)	24 (50.0%)	**0.023**
N1–3	77	23 (29.9%)	54 (70.1%)	
M stage^b^				
M0	89	34 (38.2%)	55 (61.8%)	0.827
M1	36	13 (33.3%)	23 (66.7%)	
Pathological stage^b^				
I, II	53	23 (43.4%)	30 (56.6%)	0.251
III, IV	72	24 (37.7%)	48 (62.3%)	
Recurrence				
No	32	17 (53.1%)	15 (46.9%)	**0.036**
Yes	93	30 (32.3%)	63 (67.7%)	
Grade				
I, II	95	38 (40.0%)	57 (60.0%)	0.324
III	30	9 (30%)	21 (70%)	

a *P* values were derived with a two-sided Pearson chi-square test. SD represents standard deviation.

b Tumor stage, lymph node status, and metastasis status were classified in accordance with the international system for staging lung cancer.

Finally, we examined the relationship between GIT1 expression and the clinicopathologic characteristics of NSCLC (Table 1) and found that a high level of GIT1 was positively correlated with lymph node metastasis (*P* = 0.023) and early recurrence (*P* = 0.036).

### GIT1 promotes the migration and invasion abilities of NSCLC cells

**Figure 2:**
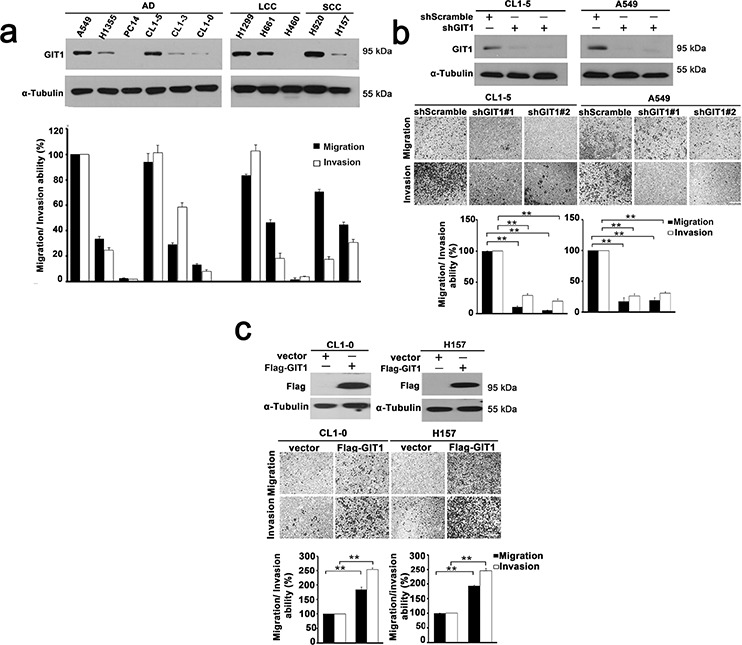
GIT1 promotes migration and invasion of NSCLC cells **a.** GIT1 expression among non-small cell lung cancer cell lines was analyzed by western blotting (Top). Quantitative data of migration and invasion of NSCLC cell lines is shown by histogram and the fold differences are compared with A549 (Bottom). AD, adenocarcinoma; LCC, large cell carcinoma; SCC, squamous carcinoma. **b.** Knockdown of GIT1 by two different GIT1-specific shRNAs in cell lines with high GIT1 expression, CL1–5 and A549. Western blot analysis shows two different shRNAs against GIT1 in CL1–5 and A549 with shScramble is used as a control. Quantitative data of migration and invasion in GIT1-knockdown CL1–5 and A549 is shown by histogram and the fold differences are compared with control cells. **c.** Overexpression of GIT1 in cell lines with low GIT1 expression, CL1–0 and H157. Western blot analysis shows expression levels of GIT1 in CL1–0 and H157, Vector, was used as a control. Quantitative data of migration and invasion of GIT1-overexpression CL1–0 and H157 are shown by histogram and the fold differences are compared with control cells. *P < 0.05. **P < 0.01.

Our clinical findings suggested that GIT1 may play an important role in NSCLC progression. We then evaluated the functional role of GIT1 on the invasiveness of lung cancer cells. Our results showed that GIT1 protein levels were significantly elevated in A549, CL1–5, H520 and H1299 cells, and that the expression of GIT1 protein was positively correlated with migration and invasion in 6 human adenocarcinoma (AD) cell lines and 5 other lung cancer cell lines including squamous cell carcinoma (SCC) and large cell carcinoma (LCC) (Figure 2a).

To determine whether GIT1 modulates lung cancer cell migration and invasion, we silenced GIT1 in A549 and CL1–5 cells using GIT1-specific lentiviral shRNAs. Our results showed that GIT1 shRNAs significantly reduced GIT1 protein with a concomitant inhibition of migration and invasion of approximately 85–90% (Figure 2b). Complementarily, overexpression of GIT1 in poorly invasive CL1–0 and H157 cells significantly enhanced their migration and invasion activity by 1.6-fold (*P* < 0.05) and 2-fold (*P* < 0.05), respectively (Figure 2c). *In vitro* proliferation assays were also performed to examine the effect of GIT1 on cell proliferation during mobility assays. The data indicated that GIT1 didn't influence the proliferation ability in NSCLC cell within 72 h (Supplementary Figure S4).

### GIT1 overexpression enhances the lung colonization and metastasis of lung cancer cells

**Figure 3:**
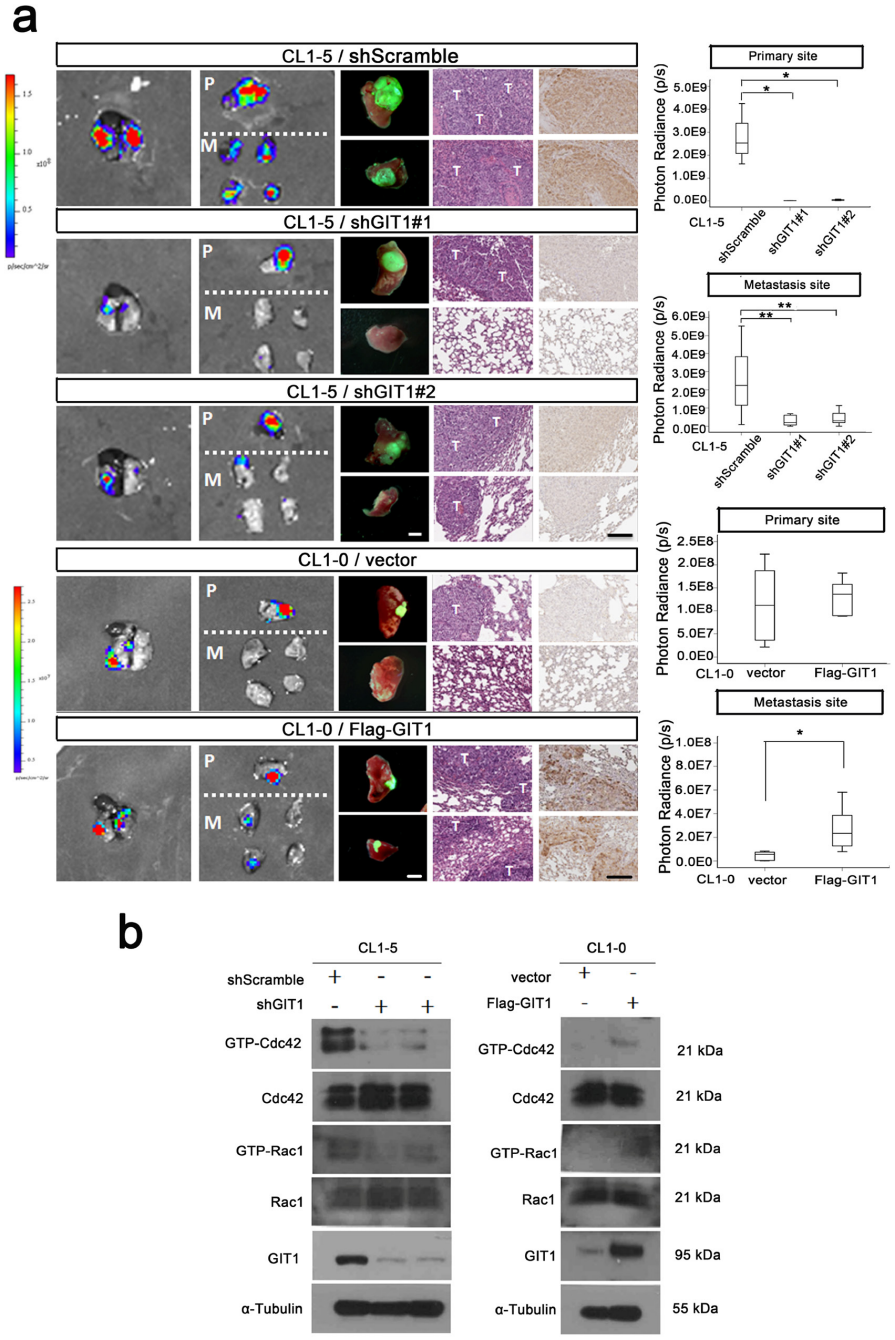
GIT1 regulates tumor growth and metastasis in orthotopic animal models We generated the CL1–5-GL and CL1–0-GL cell, which were stably expressing GFP and luciferase proteins and could detect the GFP and Luciferase signal simultaneously. We performed *in vivo* orthotopic model experiments by injecting CL1–5 and CL1–0 cells (1 × 10^6^) with or without GIT1 knockdown or expression into NOD/Shi-scid/IL-2Rγnull (NSG) mice at the left side lung and determined the metastasis to contralateral lung. **a.** Establish GIT1 knockdown CL1–5/shGIT1 and GIT1 overexpression CL1–0/Flag-GIT1 cells as described in Figure 2b. shScramble is CL1–5 group control; Vector is CL1–0 group as control. Representative photon images of lungs were taken 4–6 weeks after orthotopic injection of the indicated CL1–5 and CL1–0 cells into NSG mice. Mice were subjected to luciferase imaging (Left). GFP signaling (Middle), Representative mice lungs and H&E and GIT1 IHC staining were shown in each group (Right). Scale bar: 100 μm. The photon signals of lung metastases were quantified in each group (Primary and metastasis site). *P < 0.01. **P < 0.001; *n* = 8 mice per group. Abbreviations: *P*, primary site; M, Metastasis Site; T: Tumor site. **b.** *In vivo* orthotopic mice Rac1/Cdc42 activity assay.

We next examined the *in vivo* effects of GIT1 expression on tumor growth and metastasis. We performed *in vivo* orthotopic model experiments by injecting CL1–5 cells (1 × 10^6^) with or without GIT1 knockdown into NOD/Shi-scid/IL-2Rγnull (NSG) mice at the left side lung and determined the metastasis to contralateral lung. Decreased lung metastasis nodules was observed in the right lung of GIT1 knockdown group compared with the corresponding non-silenced control (shScramble) cell-injected groups (*P* = 0.006 and 0.004, Figure 3). Quantification of the metastatic signal in the lung tissues indicated a reduction of lung metastases of approximately 80% in mice carrying GIT1-knockdown cells compared with the control (*P* < 0.001) (Figure 3a). Besides, GIT1 knock down group also decreased the GIT1 expression and tumorigenesis ability. Similarly, control CL1–5 cells (CL1–5/shScramble) intravenously injected into NSG male mice formed large tumors after 6 weeks, while mice injected with CL1–5 cells (1 × 10^6^) expressing shGIT1 (CL1–5/shGIT1) formed small tumors. The control cells formed an average of 18 ± 6 colonies per lung compared with an average of 3 ± 2 in the CL1–5/shGIT1 group (Supplementary Figure S5a). Furthermore, similar results were also observed in lung cancer A549 xenografts (Supplementary Figure S5c).

Conversely, we also performed *in vivo* orthotopic model experiments by injecting CL1–0 cells (1 × 10^6^) with or without GIT1 overexpression into NSG mice as described above. In a comparative experiment, in which tumor metastases were rarely seem in the right lung of the mice carrying the primary tumors generated from control CL1–0 cells, enhanced lung metastasis nodules were frequently observed in the right lung of GIT1 overexpression group (*P* = 0.002, Figure 3a). Similarity, control CL1–0 cells (CL1–0/vector) intravenously injected into NSG male mice formed large tumors after 8 weeks. When mice were injected with CL1–0 cells ectopically expressing Flag-GIT1, they also grew large tumors and exhibited increased metastasis (Supplementary Figure S5b). Quantification of the metastatic nodules in the lung tissues confirmed that the number of lung metastases was significantly higher in mice carrying Flag-GIT1 cells compared with the control (*P* < 0.001). The complementary result was also achieved in lung cancer H157 xenografts (Supplementary Figure S5d).

### GIT1 regulates the activation status of Cdc42/Rac1

Rho family GTPases such as Rac1, Cdc42, and RhoA play key roles in cell migration events and in controlling actin dynamics [26]. There is some evidence that GIT1 proteins might regulate Rac1 and Cdc42 activity through construction of PIX-GIX-Paxillin protein complex [12]. Here in our results, we have found GIT1 expression levels were positively correlated with activation status of Cdc42 and Rac1 in NSCLC cell lines (Supplementary Figure S6a). Next we examined the Spearman's rho correlation to determine the GIT1 and Rac1/Cdc42 correlation in our lung cancer cohort. The correlation between GIT1 and Cdc42 is 0.396 (*P* < 0.001) and the correlation between GIT1 and Rac1 is 0.192 (*P* = 0.044) (Supplementary Figure S6b and Supplementary Table S4).

**Figure 4:**
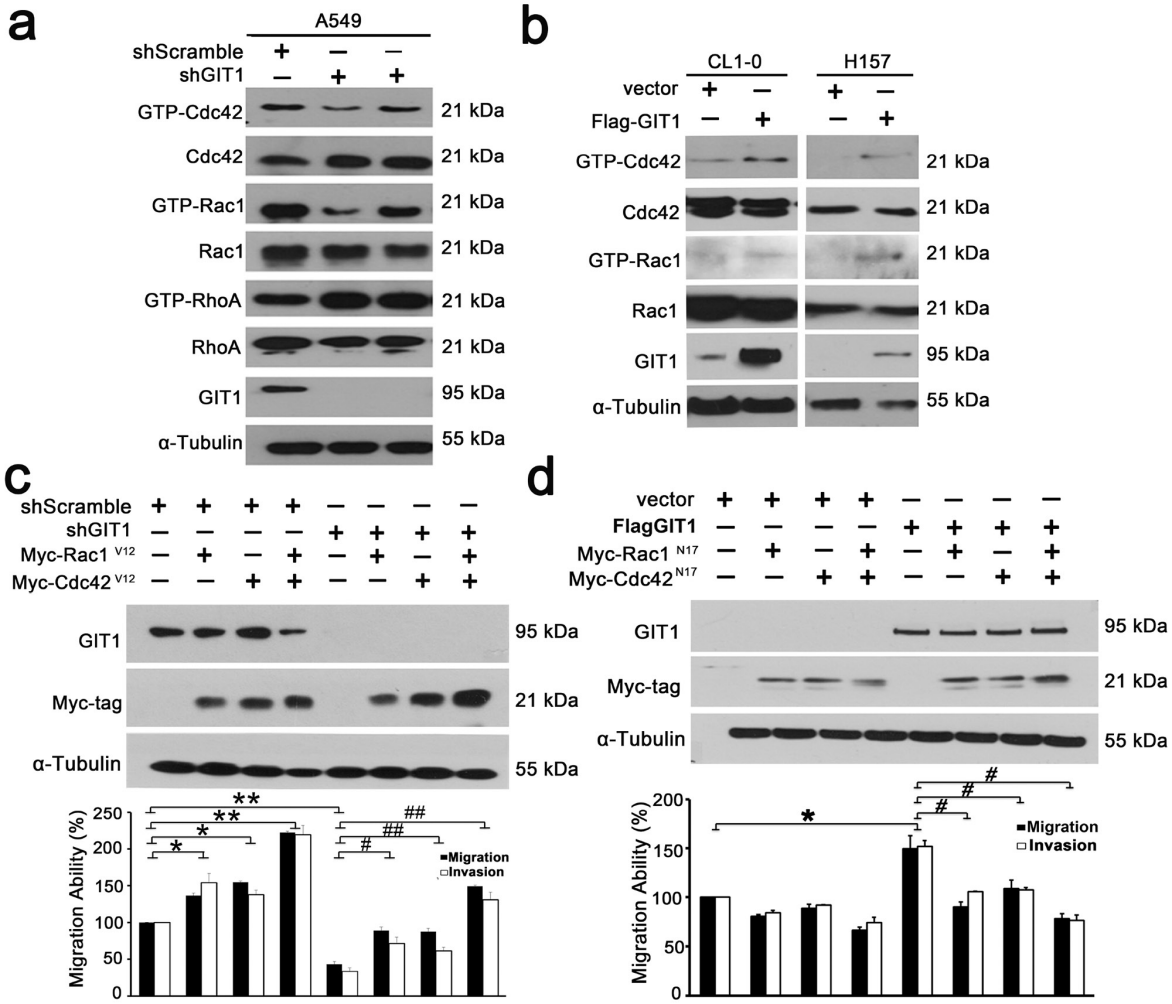
GIT1 regulates the activation status of Cdc42/Rac1 **a.** Representative blots of Rac1/Cdc42/RhoA assays. A549 cells were infected with lentivirus of shRNAs against GIT1 and with the corresponding controls as indicated. **b.** H157 and CL1–0 cells were transfected with Flag-GIT1 and with the corresponding controls. GTP-Cdc42 and Rac1 were assayed using GST-PBD. GTP-RhoA was assayed using GST-TBD. Each experiment was repeated three times and the amounts of GTP-Cdc42/Rac1/RhoA were analyzed by densitometer. **c.** Analysis of cell migration of CL1–5 cells transiently transfected with either GIT1 shRNA or control vector, or together with Myc-tagged Cdc42^V12^ or Rac1^V12^. Quantitative data of migration and invasion of CL1–5 cells for each group are shown by histogram and the fold differences are compared with control cells (left panel). *P < 0.05. **P < 0.01, ^#^*P* < 0.05. ^##^*P* < 0.01. **d.** Analysis of cell migration of H157 cells transiently transfected with either Flag-GIT1 or control vector, or together with Myc-tagged Cdc42^N17^ or Rac1^N17^. Quantitative data of migration and invasion of H157 cells for each group are shown by histogram and the fold differences are compared with control cells (left panel). *P < 0.05, ^#^*P* < 0.05.

We next examined whether the function of GIT1 in cell migration is mediated through the Rho family of GTPases by comparing levels of GTP-bound RhoA, Rac1, and Cdc42 in the A549 and H157 cell lines. A549 cells transfected with shGIT1 had lower levels of GTP-bound Rac1 and Cdc42 when compared with controls, while CL1–0 and H157 cells transfected with Flag-GIT1 had higher levels of GTP-bound Rac1 and Cdc42 (Figure 4a, 4b). We also determine the Rac1 and Cdc42 activity in xenografts from our *in vivo* orthotopic models. The data also indicated that GIT1 could regulate the activation status of Cdc42/Rac1 *in vivo* (Figure 3b). To elucidate the importance of decreased GTP-bound Cdc42 and Rac1 in GIT1-regulated cell motility, we expressed constitutively active Cdc42 (myc-Cdc42-V12) and/or Rac1 (myc-Rac1-V12) in CL1–5 cells with shGIT1 expression. Both Cdc42^V12^ and Rac1^V12^ reversed the effect of shGIT1, restoring migration to normal levels (*P* < 0.05). In addition, co-expression of Cdc42^V12^ and Rac1^V12^ went beyond reversing the effect of shGIT1, resulting in migration and invasion levels significantly higher than normal. (*P* < 0.01) (Figure 4c and Supplementary Figure S7). Furthermore, we also expressed dominant negative Cdc42 (myc-Cdc42-N17) and/or Rac1 (myc-Rac1-N17) in H157 cells with Flag-GIT1 expression. Both Cdc42^N17^ and Rac1^N17^, either alone or in combination, restored Flag-GIT1-enhanced migration back to normal levels (Figure 4d and Supplementary Figure S7). Our results suggest that GIT1-mediated regulation of lung cancer cell motility depends, at least in part, on its ability to modulate Cdc42 and Rac1 activity.

Recently, regulators of PIX/GIT1 complex were also identified to involve in regulating cell mobility. Sorting nexin family member 27 (SNX27) and Myosin XVIIIA (MYO18A) were reported to participate in regulating the intracellular transport and localization of PIX/GIT1 complex [27–29]. We have therefore evaluated whether SNX27 and MYO18A also regulate lung cancer cell motilities through the PIX/GIT1 complex. We first found that knock down of SNX27 and MYO18A expression significantly suppressed CL1–5 cell migration abilities (Supplementary Figure S8a). Next, overexpression of Flag-GIT1 failed to increase migration abilities in cells expressing shMYO18A or shSNX27 (Supplementary Figure S8b). These results indicated that MYO18A and SNX27 may serve as the upstream regulators of GIT1 in our lung cancer models. Together, our data suggest an important role of GIT1 signaling axis in the regulation of cell mobility and tumor metastasis in NSCLC.

## DISCUSSION

Lung cancer metastasis is still the main cause of cancer related death; therefore, identification of novel prognostic markers and therapeutic targets for the diagnosis and treatment of lung cancer metastasis is crucial. Previous studies have found GIT1 to be overexpressed in many types of metastatic tumors, including oral, cervical, breast, liver and colon cancers [15–18]. However, the clinical significance of GIT1 in cancer prognosis and patient survival remains unclear. In this study we investigated the role of GIT1 in lung cancer by analyzing its immunohistochemical expression in clinical NSCLC patients and its phenotypic impact *in vitro* and *in vivo*. We found high protein levels of GIT1 to be associated with shorter survival in NSCLC patients. We also identified GIT1 as an independent prognostic factor in NSCLC through multivariate regression analysis. To our knowledge, our study provides the first evidence suggesting that GIT1 protein might be a viable prognostic marker for NSCLC, especially in prediction of clinical outcome in early stage lung cancer patients. In addition, we determined that GIT1 regulates the invasiveness of NSCLC cells by altering Rac1/Cdc42 activity.

Several studies have revealed the involvement of GIT1 in the development and progression of cancer, including cell transformation, growth and migration. For example, it has been shown that methionine adenosyltransferase 2B (MAT2B) and GIT1 form a complex to control cancer cell growth and are overexpressed in most human liver and colon cancer specimens [18]. It is also worth noting the significant correlation we found between GIT1 and tumor grade and progression. Yoo *et al*. have shown GIT1 expression to be correlated with tumor grade in cervical cancer and Wang *et al*. have shown higher GIT1 expression to be correlated with advanced grades of oral squamous cell carcinoma (OSCC) and lymph node metastasis of OSCC [15, 16]. In our study, although no significant correlation between GIT1 expression and tumor grade of lung cancer was found, GIT1 proteins were overexpressed in lung cancer tissue but not in non-cancerous lung tissue and higher GIT1 expression was correlated with poor prognosis. As such, our study is the first to demonstrate that GIT1 expression might serve as a predictive marker for lung cancer progression.

As a multi-functional scaffold protein, GIT1 binds to diverse signaling proteins to play an important role in a wide range of regulatory processes, especially in cell mobility. GIT1 has been shown to form a complex with paxillin, PIX and PAK in focal adhesions to regulate cell migration. PIX-GIT1-Paxillin forms a pro-migratory protein complex that mediates Rac1 and Cdc42 (Rho family of GTPase) activation in the focal complexes of leading edges [8]. In recent years, the Rac1/Cdc42 pathway has been extensively studied and implicated in cancer metastasis and EMT-mediated cell migration and invasion [30–35]. Rac1/Cdc42 overexpression also has been correlated with poor clinical outcome in NSCLC and other cancers [30, 32–35]. GIT1 expression also associated with poor prognosis in multiple cancers, including cervical, breast, OSCC, HCC and colon cancers. This raises the possible correlations and importance between GIT1 and Rac1/Cdc42 in regulating cancer progression. Although our data provide potential relationship of GIT1 and Rac1/Cdc42 in Figure 2 and Supplementary Figure S4. But it doesn't have higher correlation level in our lung cancer cohort. It is indeed difficult to determine the activation status of Rac1/Cdc42 with paraffin-embedded specimens. We can only determine the total protein but not active form of Rac1/Cdc42 in our clinical samples. Further evaluations with fresh specimens may be needed to confirm their clinical correlation. According to our *in vitro* functional assays, GIT1 regulates cancer cell mobility through Rac1/Cdc42 activation in NSCLC cells. Moreover, both Cdc42^V12^ and Rac1^V12^, the constitutive forms of Rac1 and Cdc42, reversed the effect of shGIT1-suppressed motility. Conversely, both Cdc42^N17^ and Rac1^N17^, the dominant negative forms of Rac1 and Cdc42, abolished the effect of GIT1-enhanced motility. Our data thus suggests that Rac1/Cdc42 activation plays an important role in GIT1-induced invasiveness of NSCLC cells. We also speculate that GIT1 may serve as a master switch for Rac1/Cdc42 activity in NSCLC cells.

It is known that tissue micro-environment may alter metastatic abilities of cancer cells. Therefore, in contract to subcutaneous and tail vail injection, orthotropic model is obviously better way to mimic metastasis situation. Our orthotopic metastatic model demonstrated that GIT1 expression is important in promoting cancer cell metastasis from the primary sites. However, since the primary tumor growth was also affected by GIT1 manipulations, especially GIT1 depletion, our data still cannot rule out the effects of metastasis from difference of primary tumor size. Recently, surgical orthotopic implantation (SOI) of histologically-intact cancer fragments have been recognized as a better way to promote cancer metastasis in the transplanted mice and reflect more of the clinical cancer pattern [36–40]. It may be a better model for evaluating metastatic roles of GIT1 in NSCLC and also avoid the effects from primary tumor size in the future. Furthermore, improving sensitivity and resolution of fluorescent protein imaging may also provide more information about the local invasion and metastatic behavior affected by GIT1 [41–44]. Collectively, although further experiments may be needed, our *in vivo* animal models, including experimental and orthotopic metastatic models, suggested an important role of GIT1 in regulating metastasis of NSCLC cells.

In addition, GIT1 has also been found to localize with focal adhesion kinase (FAK) and paxillin at focal adhesion points to promote cell motility. [12] FAK is a positive regulator of tumor invasion and migration, and is overexpressed in many cancers, including breast, cervical, colon, liver and NSCLC [45, 46]. Previous research has shown that miR-491–5p targets GIT1 to inhibit OSCC cell focal adhesion formation, invasion and metastasis through regulation of FAK and paxillin. [15] Furthermore, it has been reported that GIT1 stabilizes integrin, a major upstream activator of FAK, resulting in focal adhesion formation of metastatic breast cancer cells. [17] Therefore, the levels of paxillin and FAK in GIT1-induced invasiveness of NSCLC ought to be examined in future studies.

In sum, we have demonstrated that GIT1 was overexpressed in NSCLC as compared to non-tumor lung tissues and was related to poor prognosis of NSCLC. GIT1 expression was also correlated with lung cancer progression in NSCLC. GIT1 expression increased lung cancer cell invasiveness through Rac1/Cdc42 activity and promoted tumor growth and metastasis *in vivo*. We speculate that GIT1 is a predictive marker for cancer progression in NSCLC and may be a potential therapeutic target for lung cancer patients.

## MATERIALS AND METHODS

### Case selection

A total of 125 patients diagnosed with non-small cell lung cancer at the Kaohsiung Medical University Hospital of Taiwan from 1991 to 2007 were included in this study. All patients received standard treatment protocols according to hospital guidelines. Patients with operable stage I–III NSCLC underwent lobectomy or pneumonectomy with mediastinal lymphadenectomy. No adjuvant chemotherapy was administered for patients with completely resected stage I NSCLC. Patients with resectable stage II and III NSCLC were treated with postoperative adjuvant platinum-based chemotherapy. Patients with non-resectable locally advanced or metastatic disease received chemotherapy with or without radiotherapy. Clinical information and pathology data were collected via retrospective review of the medical records. All cases were staged according to the cancer staging manual of the American Joint Committee on Cancer (AJCC) and the histological cancer type was classified according to World Health Organization (WHO) 2004 classification. Follow-up data were available in all cases, and the longest clinical follow-up time was 190 months. Overall survival (OS) and disease-free survival (DFS) were defined as the interval after treatment to death from any cause and to recurrence or distant metastasis or death, respectively. The study was carried out with the approval of the Institutional Review Boards and with permission from the ethics committees of the institution involved (KMUH-IRB-20110286). Another tissue microarray from a Korean cohort of non-small cell lung cancer patients was purchased from SuperBioChips (SuperBioChips Laboratories, Seoul, Korea).

### Public online database

The Cancer Genome Atlas (TCGA), Kaplan-Meier plotter and SurvExpress were used to analyze the role of GIT1 in clinical lung cancer patients. TCGA database contains 1124 lung cancer patients and 105 paired non-tumor lung tissue [20]. Kaplan-Meier plotter database [21] and SurvExpress database [22] we used contained multiple GSE datasets (GSE14814, GSE19188, GSE29013, GSE31210, GSE3141, GSE37745, GSE4573 and GSE8894) to evaluate the correlation between GIT1 expression and patient outcomes.

### Cell culture

Human lung cancer cell line H1355, CL1–0, CL1–5, H460, H661 and H157 were grown in RPMI 1640 supplemented with 10% FBS (Invitrogen, CA, USA) and 1% Penicillin-Streptomycin-Glutamine (Gibco, CA, USA). A549 were grown in F12K supplemented with 10% FBS and 1% Penicillin-Streptomycin-Glutamine. H1299, PC13, PC14 and H520 were maintained in DMEM supplemented with 10% FBS and 1% Penicillin-Streptomycin-Glutamine. All cells were incubated in a CO_2_ incubator containing 5% CO2 at 37°C. CL1–0 and CL1–5 were established by Chu and colleagues and displayed progressively increasing invasiveness [23]. PC14 was developed by Lee and colleagues at National Cancer Center Hospital, Tokyo, Japan [24]. Other lung cancer cell lines (A549 and H1299) were obtained from the American Type Culture Collection (ATCC).

### Tissue microarray construction and immunohistochemistry staining

Three representative 1-mm-diameter cores from each tumor taken from the formalin-fixed paraffin embedded tissues were selected by morphology typical of the diagnosis. Assessable cores were obtained in a total of 125 cases. Paired normal lung tissue samples were also obtained in 56 cases. The histopathologic diagnosis of all samples were reviewed and confirmed by two pathologists (Chia-Yi Su and Michael Hsiao) via hematoxylin and eosin-stained slides. Immunohistochemical (IHC) staining was performed on serial 5-micrometer-thick tissue sections cut from the tissue microarray (TMA) using an automated immunostainer (Ventana Discovery XT autostainer, Ventana Medical Systems, Tucson, AZ, USA). Briefly, sections were first dewaxed in a 60°C oven, deparaffinized in xylene, and rehydrated in graded alcohol. Antigens were retrieved by heat induced antigen retrieval for 30 minutes with TRIS-EDTA buffer. Slides were stained with a polyclonal rabbit antihuman GIT1 antibody at 1/100 dilution (GeneTex, Taipei, Taiwan) for 1 hour at room temperature. The sections were then labeled with HRP-conjugated secondary antibody at 1/100 dilution for 1 hour. Finally, the sections were developed with DAB substrate kit (vector lab, SK-4200) at room temperature for 5 to 8 minutes. The sections were subsequently counterstained with hematoxylin, dyhydrated, and mounted.

### TMA immunohistochemistry interpretation

The IHC staining assessment was independently conducted by 2 pathologists (Chia-Yi Su and Michael Hsiao) blinded to patient outcome. Only cytoplasmic IHC expressions of tumor cells in the cores were evaluated. Both the immunoreactivity intensity and percentage were recorded. The intensity of staining was scored using a four-tier scale and defined as follows: 0, no staining; 1+, weak staining; 2+, moderate staining; 3+, strong staining. The extent of staining was scored by the percentage of positive cells (0–100%). The final IHC scores were obtained by multiplying staining intensity by the percentage of positive cells. All cases were divided into two groups according to the final IHC scores. High IHC expression level was defined as a score greater than or equal to 150 and a score less than 150 was defined as low expression.

### Knockdown and transfection

Knockdown of GIT1, MYO18A, and SNX27 were performed by short-hairpin RNAs (shRNAs). Derivatives of shRNA vector were obtained from National RNAi Core Facility Platform and target sequences list as follows: pLKO.1-shGIT1–1: CCTGCTCAGAGA AGATCCATT (human GIT1 CDS); pLKO.1-shGIT1–2: GCTCTCCCTTTAATGCCATAT (human GIT1 3′UTR); pLKO.1-shMO18A-1:CGGAAGGAGAAGAAGGAGAAA (human MYO18A CDS); pLKO.1-shMO18A-2:CGAATTG ATGAAGAAGCACAA (human MYO18A CDS); pLKO.1- shSNX27–1:TACGTAAATTGGCACCTAATG (human SNX27 CDS); pLKO.1-shSNX27–2:CAAATTAGCTGCA CGTATATA (human SNX27 3′UTR). The procedures of lentivirus packaging and infection were followed as previously described [19]. Transient transfections of Flag-GIT1 were performed with Lipofectamine 2000 (Invitrogen, Carlsland, Ca) according to the manufacturer's instructions. The cells were cultured for 48 hours and collected for further analysis.

### Two-Chamber migration/invasion assay

Cell mobility was determined by a two-chamber migration assay (8 mm pore size, BD Biosciences). Approximately 2 × 10^4^ cells were seeded into the upper chamber and allowed to migrate into the lower chamber for 18–24 hours. Cells in the upper chamber were carefully removed using cotton buds and cells at the bottom of the membrane were fixed and stained with 0.2% crystal violet/20% methanol. Quantification was performed by counting the stained cells. The migrated cells were counted under a light microscope (200 folds, 5 random fields from each well). All experiments were performed in triplicate. For the invasion assay, 8-mm Polycarbonate filters were coated with Matrigel on the lower side. Approximately 2 × 10^5^ cells were loaded on the upper chamber. After 18–24 hours, the membranes were fixed and stained with 0.2% crystal violet/20% methanol. Quantification was performed by counting the stained cells. The invaded cells were counted under a light microscope (200 folds, 5 random fields from each well). All experiments were performed in triplicate.

### Rac1/Cdc42 activity assay

Rho/Rac/Cdc42 Activation Assay Combo Kit was used to perform Rac activation assay as described previously [19]. Briefly, cells were washed in cold PBS and then lysed in buffer B (50 mM Tris [pH 7.5], 1% Triton X-100, 150 mM NaCl, 5 mM MgCl2, 1 mM DTT, 1 mM PMSF, 10 μg/mL aprotinin, and 1 μg/mL leupeptin). GTP-bound Rac1 and GTP-bound Cdc42 were affinity precipitated from cell lysates (1 to 1.5 mg of protein) using an immobilized GST fusion construct of the Rac1 binding domain of Pak (the p21Rac binding domain [PBD]) that binds to Rac1-GTP but not to Rac1-GDP [25] for 30–45 minutes. The complexes were then subjected to western blot analysis using Rac1-specific and Cdc42-specific antibodies. Total cellular lysates were also separated by SDS-PAGE, and western blot analysis with anti-Rac/Cdc42 antibodies was done as a control for protein loading. Essentially the same assay was used to measure RhoA-GTP, except that for these assays the RhoA binding domain (RBD) was used as a GST construct. RhoA that sedimented with the GST-RBD beads was detected with an antibody against RhoA.

### Western blot analysis

The protein lysates (30–50 μg/sample assessed by BCA protein assay, Pierce Chemical Co., IL) were subjected to 10% SDS-Tris glycine gel electrophoresis and transferred to polyvinylidene difluoride (PVDF) membranes (Millipore, USA). The membranes were incubated with polyclonal GIT1 antibody (Cell Signaling, 1:1000), Rac1 antibody (Millipore 1:1000), Cdc42 antibody (Cell Signaling, 1:1000), RhoA antibody (Cell Signaling, 1:1000), MYO18A antibody (Santa Cruz. 1:1000), SNX27 antibody (Santa Cruz. 1:1000) and α-tubulin (Cell Signaling, 1:5000), at 4°c overnight. Peroxidase conjugated anti-rabbit antibody (1:5000) was used as a secondary antibody. The membranes were developed by Renaissance protein detection kit (DuPont NEN, MA, USA).

### Animal studies

All animal work was performed at the Genomics Research Center of Academia Sinica in accordance with protocols approved by Academia Sinica Institutional Animal Care and Utilization Committee. The cells (1 × 10^6^ for each group) immersed in 0.1 ml of PBS were injected into NOD/Shi-*scid*/IL-2Rγ^null^ (NOG) male mice (6–8 weeks old) via tail vein. After 4 weeks, all mice were euthanized and their lungs were removed for further photon imaging or H&E staining. Luciferase activity of mice lungs was measured by an IVIS Spectrum imaging system (Caliper Life Sciences, Hopkinton, MA). The mice lung tissues were also fixed with formalin, embedded with paraffin, sectioned and then stained with hematoxylin and eosin (H&E). The number of surface metastases per lung was counted under dissecting microscope.

### Statistical analysis

Statistics analysis was performed with SPSS 17.0 software (SPSS, Chicago, Illinois, USA). A paired *t*-test was performed to compare GIT1 IHC expression in cancer tissues and in the corresponding normal mucosal tissues. The association between clinicopathological categorical variables and GIT1 IHC expression was analyzed by Pearson's chi-square test. Survival rates were calculated using the Kaplan-Meier method and was analyzed using the log-rank test. Follow-up time was censored if the patient was lost during follow-up. Univariate and multivariate analyses were performed using Cox proportional hazards regression analysis with and without an adjustment for GIT1 IHC expression level, tumor stage, lymph node stage, and metastasis. For all analyses, *P* value < 0.05 was considered statistically significant.

## SUPPLEMENTARY FIGURES AND TABLES


