# Supplementary material for: GIT1 promotes lung cancer cell metastasis through modulating Rac1/Cdc42 activity and is associated with poor prognosis
Source: Oncotarget. 2015 Oct 8;6(34):36278–91. doi: 10.18632/oncotarget.5531 (PMC4742177; doi:10.18632/oncotarget.5531)
Supplement: Supplementary file 1 [file oncotarget-06-36278-s001.pdf]

SUPPLEMENTARY FIGURES AND TABLES

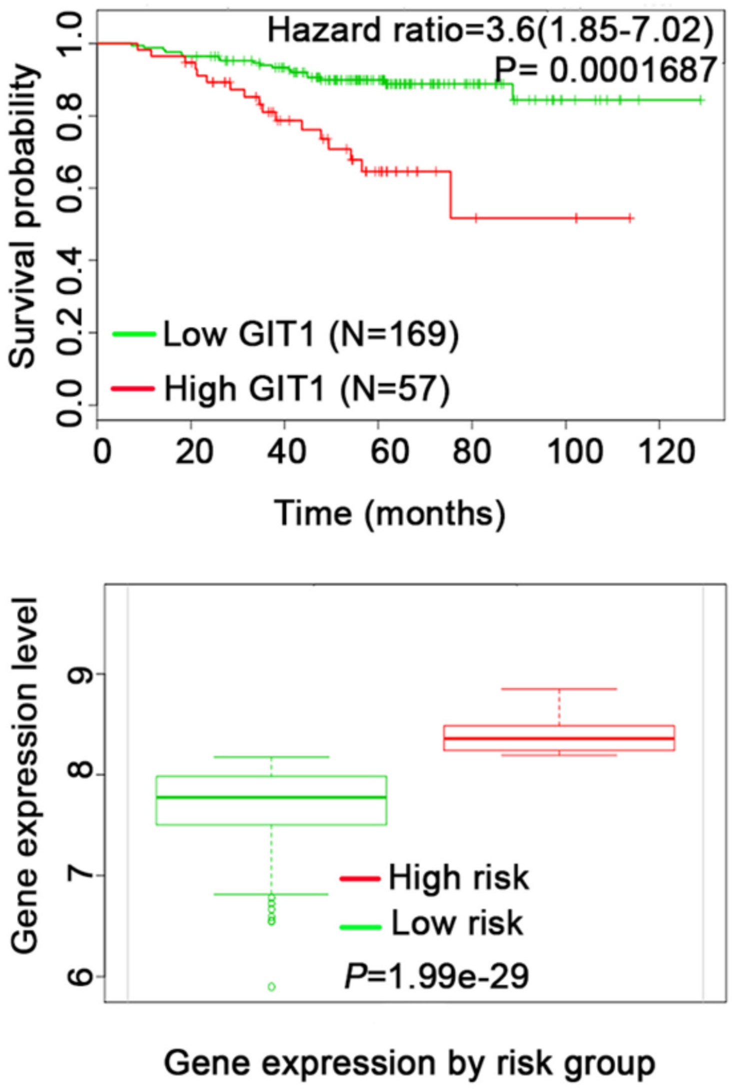

Supplementary Figure S1: Kaplan-Meier overall survival plot for patients with lung cancer. We used SurvExpress web resource, available at http://bioinformatica.mty.itesm.mx/SurvExpress, to analyze the clinical lung cancer significance of GIT1 mRNA expression in GSE31210 microarray database. Red line indicates patients with high GIT1 expression; green line indicates patients with low GIT1 expression. Individual database number and *P* value of each plot are indicated.

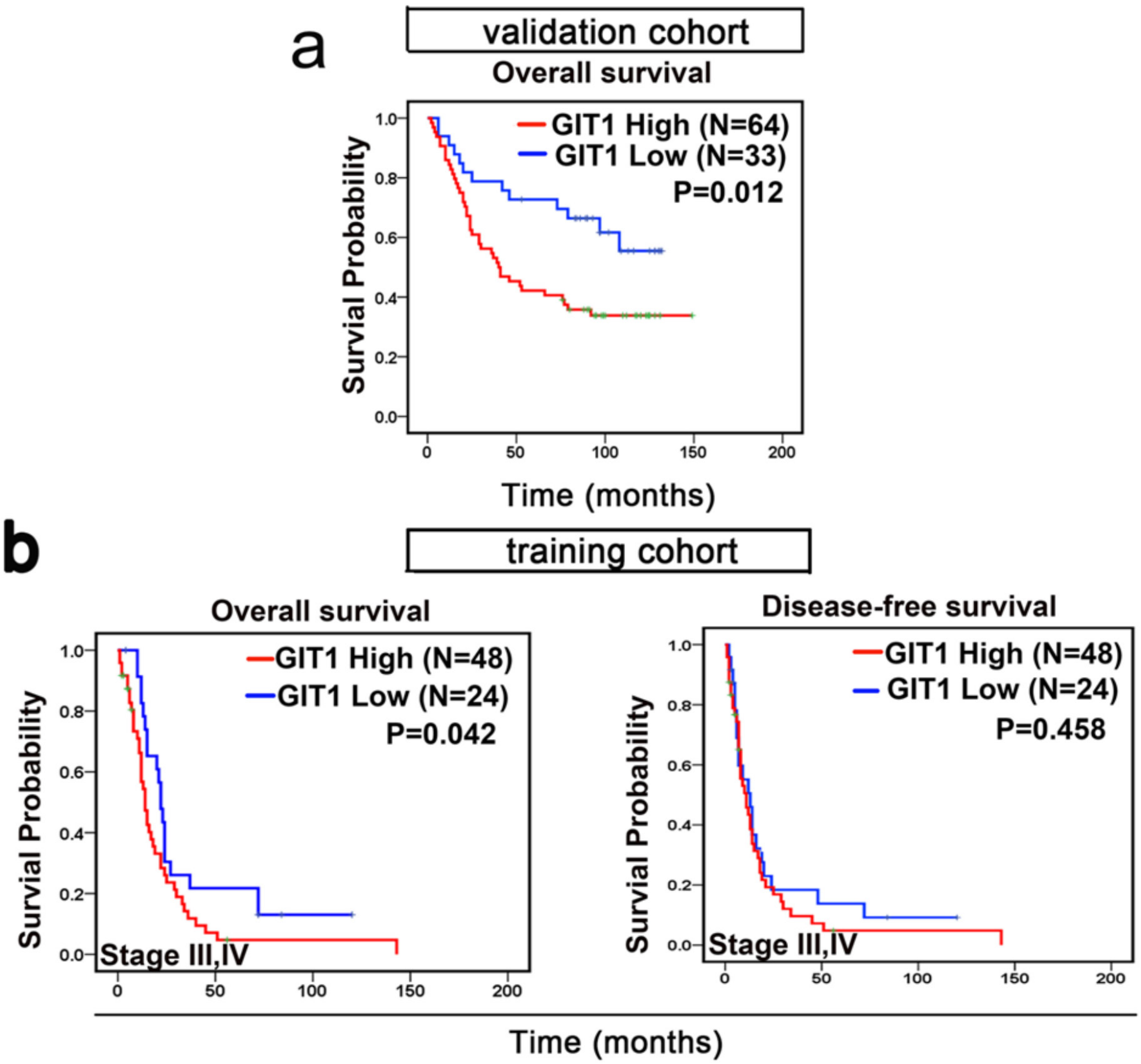

Supplementary Figure S2: GIT1 expression in lung cancer is correlated with poor prognosis and as an early lung cancer marker in non-small cell lung cancer. a. Kaplan–Meier plot of overall survival of 97 patients from the Korea cohort used as a validation set with lung cancer stratified by GIT1 level. **b.** We classified 125 patients with NSCLC at the Kaohsiung medical university Hospital of Taiwan into early stage (stages I, II) and late stage (stages III, IV). **b, c.** Kaplan–Meier plot of overall survival of late stage non-small cell lung cancer stratified by GIT1 level. The differences between groups were tested using log rank tests.

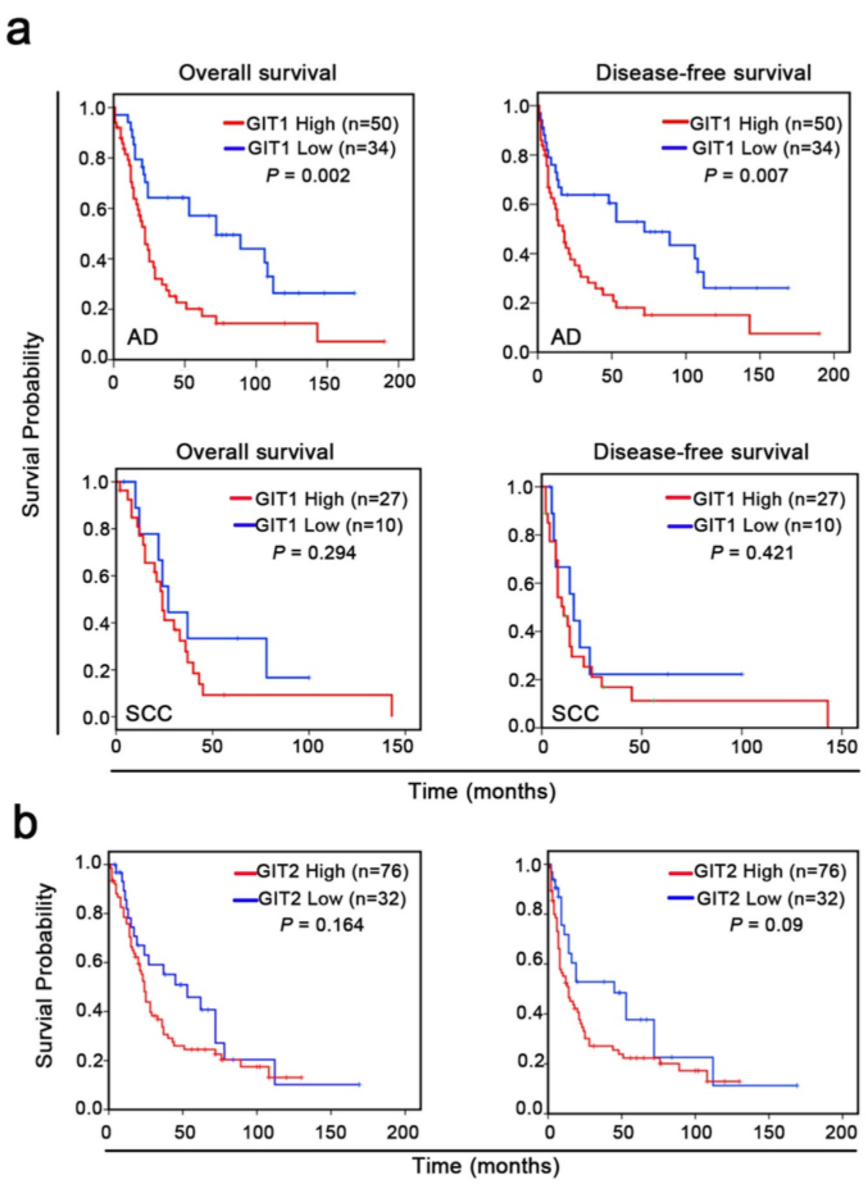

Supplementary Figure S3: GIT1 expression correlated with poor survival of NSCLC especially in adenocarcinoma. **a.** Training cohort of NSCLC patients was classified to adenocarcinoma (AD) and squamous cell carcinoma (SCC). Kaplan–Meier plot of overall survival of AD and SCC were stratified by GIT1 level. The differences between groups were tested using log rank tests. **b.** Kaplan–Meier plots of overall survival and disease-free survival of 125 patients (training cohort) with NSCLC were stratified by GIT2 level. The differences between groups were tested using log rank tests.

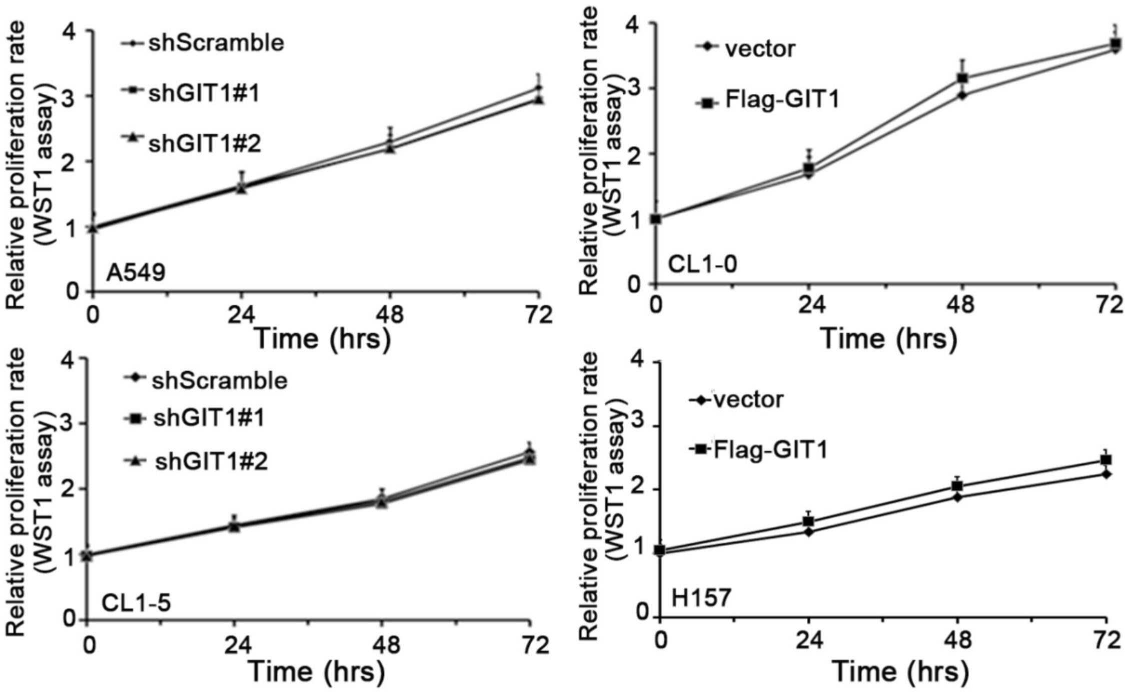

Supplementary Figure S4: GIT1 didn't influence the proliferation ability in NSCLC cell within 72 h. GIT1 knock down in CL1–5 and A549 cells and Flag-GIT1 overexpression in CL1–0 and H157 cells were subjected to WST1 assay for three days. Proliferation rates were normalized and estimated compare to their individual start point (day 0).

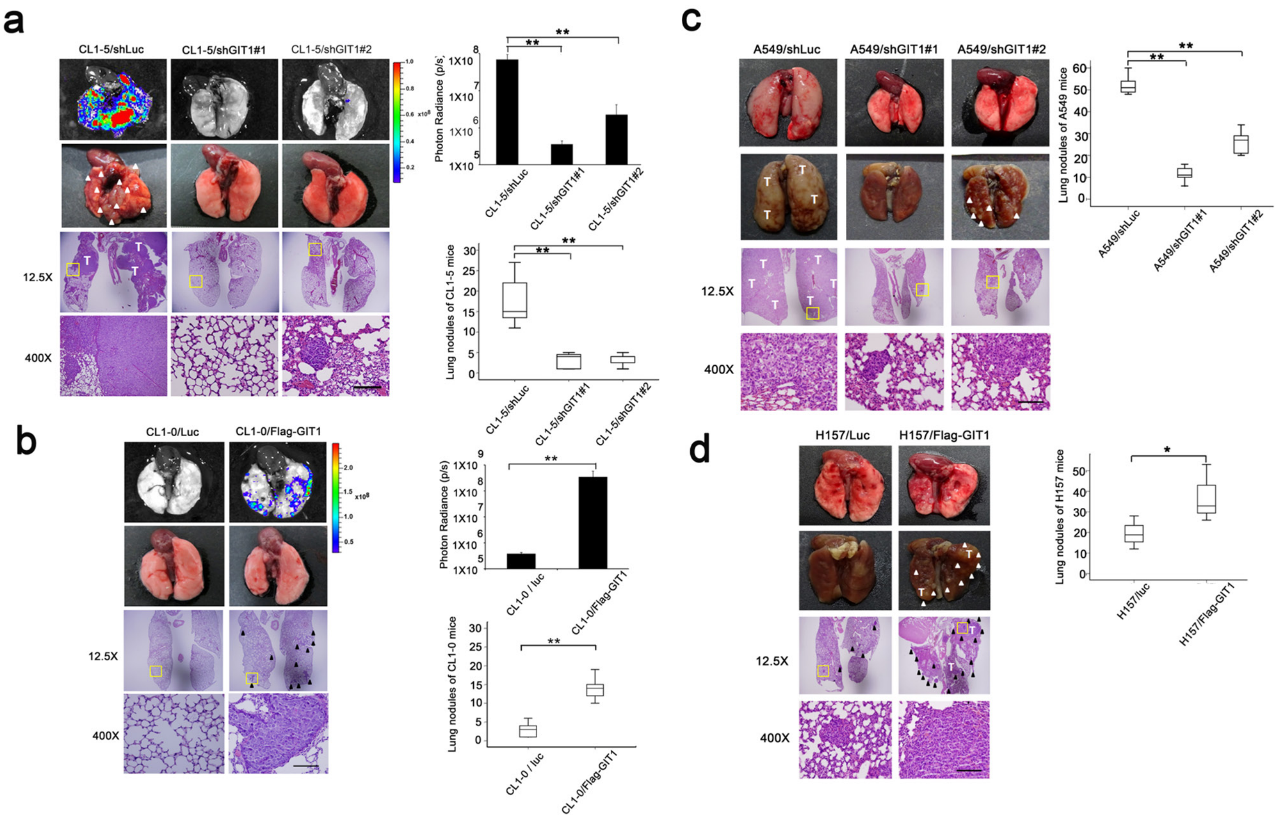

Supplementary Figure S5: GIT1 regulates tumor growth and metastasis *in vivo*. a. Establish GIT1 knockdown CL1–5/shGIT1 cells as described in Figure 2b. shScramble is as control. Representative photon images of lungs were taken 6 weeks after intravenous injection of the indicated CL1–5 cells into NOD/Shi-*scid*/IL-2Rγ^{null} (NSG). Mice were subjected to luciferase imaging (Upper). Representative mice lungs and H&E staining were shown in each group (Middle and lower). Scale bar: 100 µm. The photon signals of lung metastases were quantified in each group (upper). Numbers of surface metastases in lungs of mice were counted in each group (lower). **b.** Establish GIT1 overexrpession CL1–0/Flag-GIT1 cells as described in Figure 2c. Vector is as control vector. Representative photon images of lungs were taken 6 weeks after intravenous injection of the indicated CL1–0 cells into NSG mice. Mice were subjected to luciferase imaging (upper). Representative mice lungs and H&E staining shown for each group (Middle and lower). Scale bar: 100 µm. The photon signals of lung metastases were quantified for each group (upper). Numbers of surface metastases in lungs of mice were counted for each group (lower). $^*P < 0.01$. $^{**}P < 0.001$; $n = 6$ mice per group. **c.** GIT1 knockdown A549/shGIT1 cells were established as described in Figure 2b with shScramble serving as a control. The condition is follow as CL1–5 group. Mice were subjected to luciferase imaging (Upper). Representative mice lungs and H&E staining are shown for each group (Middle and lower). Scale bar: 100 µm. **d.** Establishment of GIT1 overexpression in H157/Flag-GIT1 cells as described in Figure 2c with vector serving as a control vector. The condition is follow as CL1–0 group. Mice were subjected to luciferase imaging (upper). Representative mice lungs and H&E staining are shown for each group (Middle and lower). Scale bar: 100 µm.

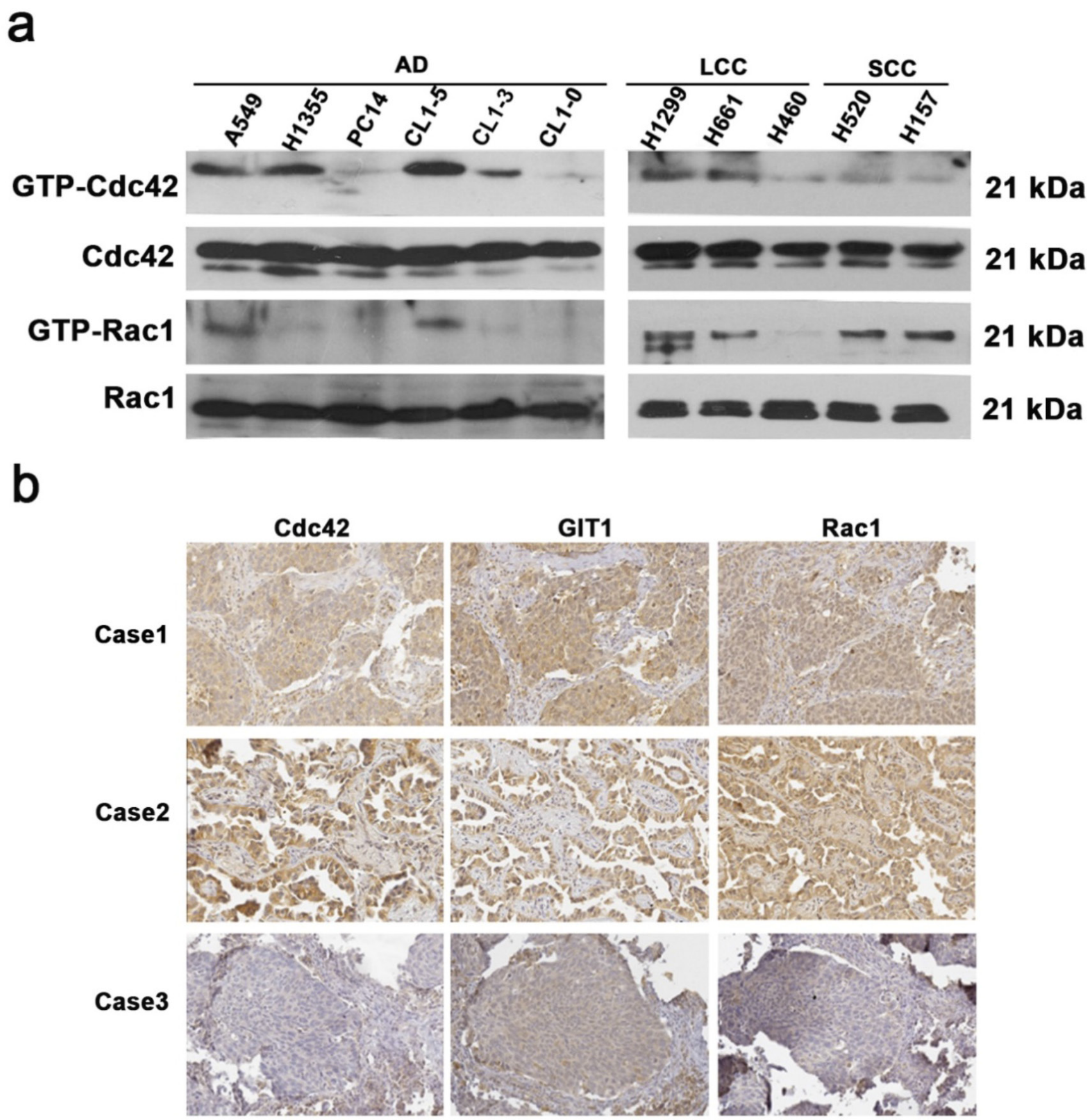

Supplementary Figure S6: GIT1 expression is positively correlated with activation of Cdc42 and Rac1. a. GIT1 protein levels were significantly elevated in A549, CL1–5, H520 and H1299 cells, and that the expression of GIT1 protein was positively correlated with activation of Cdc42 and Rac1 **b.** GIT1, Cdc42 and Rac1 protein correlation determined by IHC staining.

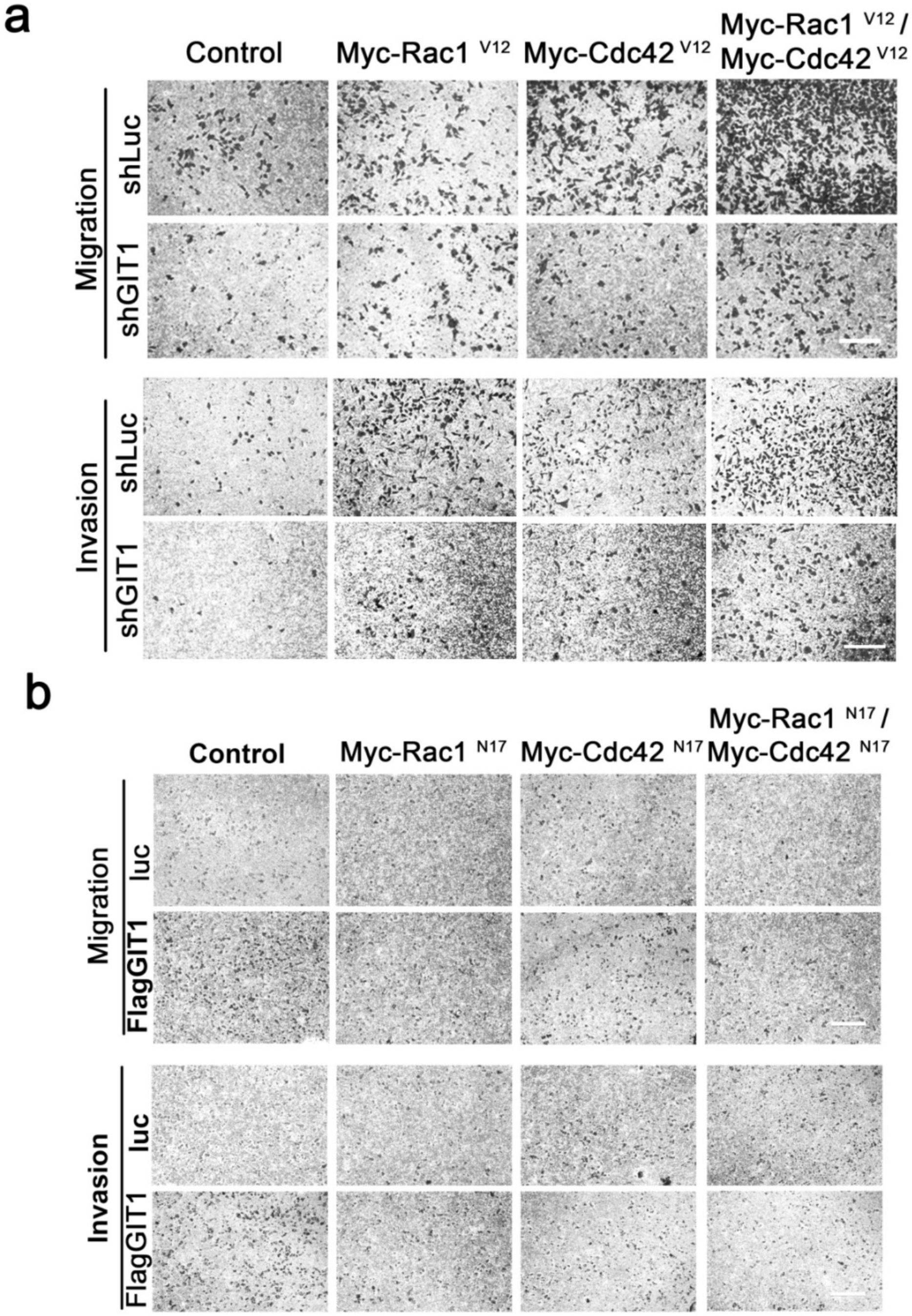

Supplementary Figure S7: GIT1 alters activation of Cdc42/Rac1. Representative images of migration and invasion of cells for each group were observed by microscopy (200x). **a.** Analysis of cell migration of CL1–5 cells transiently transfected with either GIT1 shRNA or a control vector, or together with Myc-tagged $Cdc42^{V12}$ or $Rac1^{V12}$. **b.** Analysis of cell migration of H157 cells transiently transfected with either Falg-GIT1 or a control vector, or together with Myc-tagged $Cdc42^{N17}$ or $Rac1^{N17}$.

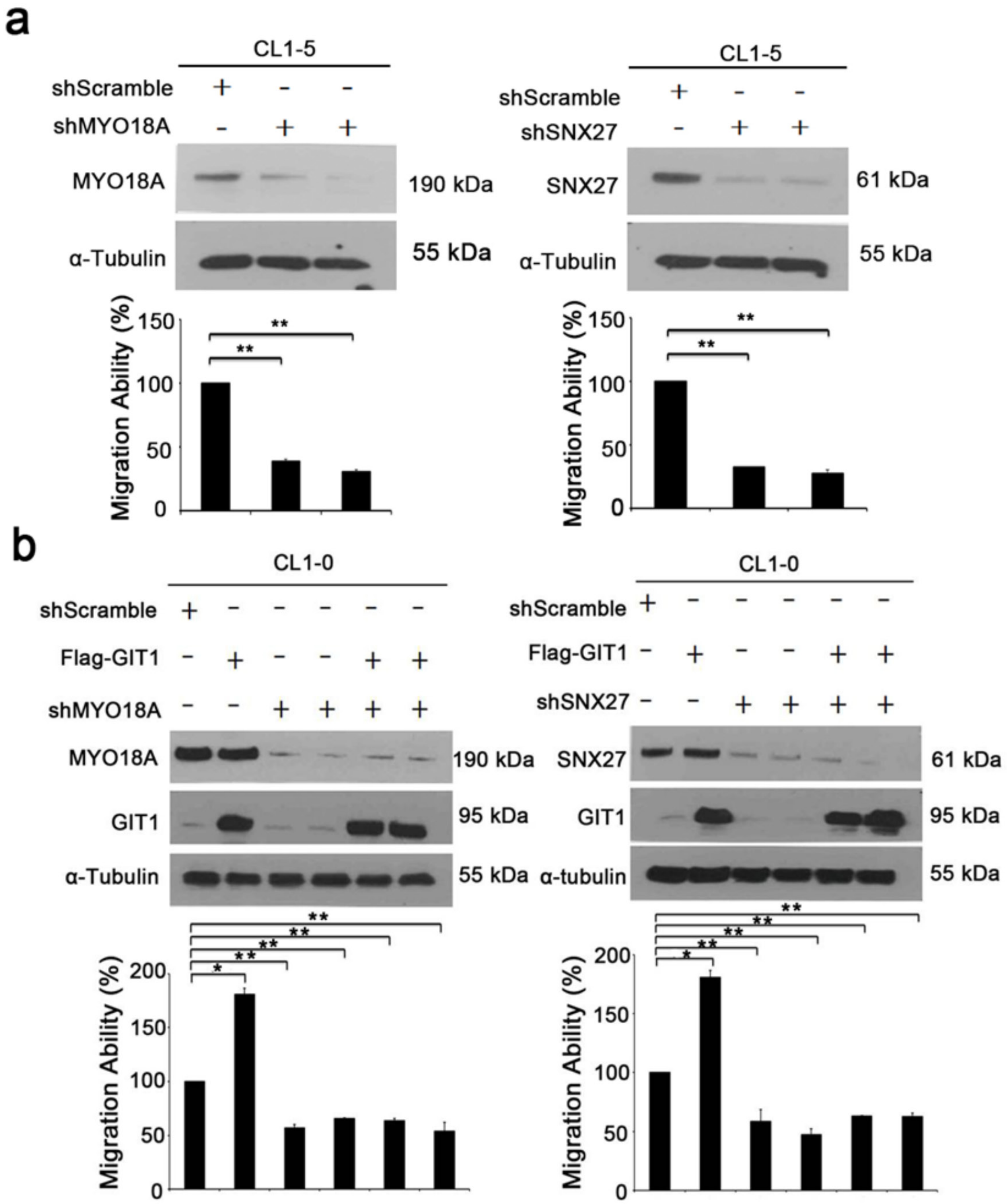

Supplementary Figure S8: MYO18a and SNX27 participate in GIT1 regulated NSCLC cell mobility. a. CL1–5 cells were infected with MYO18A- or SNX27-specific shRNAs and subjected to transwell migration assay and Western blot analysis. Migration ability was compared with cells expressing Scramble shRNA. **b.** MYO18A or SNX27 expression was both depleted by two target-specific shRNAs in CL1–0 cells. Scramble shRNA was also used as controls. Western blot analysis shows the knock down efficiency of shRNAs. Quantitative data of migration is shown by fold differences are compared with control cells. $*P < 0.05$. $**P < 0.01$.

Supplementary Table S1: Summary of Clinicopathologic Features of 125 Patients in the training cohort with Non-Small Cell Lung Cancer

Characteristic		Number	%
Age			
	< 65	73	58
	≧65	52	42
Gender			
	Male	71	56
	Female	54	44
Pathological stage			
	I	38	30
	II	15	12
	III	35	28
	IV	37	30
pT (primary tumor)			
	pT1	26	21
	pT2	63	50
	pT3	8	6
	pT4	28	23
pN (regional lymph node metastasis)			
	pN0	48	38
	pN1–3	77	62
pM (distant metastasis)			
	pM0	89	71
	pM1	36	29
Histological type			
	Adenocarcinoma	81	65
	Squamous cell carcinoma	35	28
	Adenosquamous carcinoma	2	2
	Large cell carcinoma	7	5
Recurrence			
	No	32	26
	Yes	93	74
Smoking			
	No	75	60
	Yes	50	40

Supplementary Table S2: Cox Univariate and Multivariate Regression Analysis of TNM Prognostic Factors and GIT1 Expression in training cohort

Characteristic	Univariate analysis	*P*	Multivariate analysis	*P*
	HR (95% CI)		HR (95% CI)	
**Overall survival**				
GIT1	2.21 (1.41–3.47)	0.001	2.35(1.46–3.79)	< 0.001
T	1.99 (1.27–3.09)	0.002	1.78(1.08–2.93)	0.023
N	2.71 (1.71–4.29)	< 0.001	1.88(1.14–3.10)	0.013
M	2.79(1.79–4.36)	< 0.001	2.22(1.37–3.13)	0.001
Grade	1.67(1.05–2.65)	0.03	1.95(1.21–3.13)	0.006
**Disease-free survival**				
GIT1	2.00 (1.28–3.14)	0.003	2.03(1.27–3.26)	0.003
T	2.10 (1.35–3.28)	0.001	1.66(1.01–2.72)	0.047
N	2.75(1.73–4.39)	< 0.001	1.98(1.20–3.27)	0.008
M	2.67(1.73–4.11)	< 0.001	1.91(1.19–3.09)	0.008
Grade	1.59 (1.00–2.53)	0.049	1.70(1.06–2.70)	0.027

NOTE: Cox proportional hazards regression was used to test independent prognostic contribution of GIT1 after accounting for other potentially important covariates. Abbreviations: HR, hazard ratio; CI, CI, confidence interval.

Supplementary Table S3: Cox Univariate and Multivariate Regression Analysis of TNM Prognostic Factors and GIT1 Expression in validation cohort

Characteristic	Univariate analysis HR (95% CI)	*P*	Multivariate analysis HR (95% CI)	*P*
**Overall survival**				
GIT1	2.17(1.16–4.05)	0.015	2.06(1.10–3.86)	0.024
T	2.18(1.16–4.98)	0.015	1.92(1.01–3.65)	0.047
N	1.95(1.15–3.33)	0.014	1.67(0.96–2.89)	0.068

NOTE: Cox proportional hazards regression was used to test the independent prognostic contribution of GIT1 after accounting for other potentially important covariates. Abbreviations: HR, hazard ratio; CI, confidence interval.

Supplementary Table S4: Spearman's rho correlation to determine the GIT1 and Rac1/Cdc42 correlation in training cohort

	GIT1	CDC42	Rac1
GIT1	1		
Cdc42	0.396 ($P < 0.001$)	1	
Rac1	0.192 ($P = 0.044$)	0.316 ($P = 0.001$)	1

NOTE: Spearman's rho correlation was used to test the correlation between of GIT1, Rac1 and Cdc42.
